#  Simultaneous Spectrophotometric Determination of Rifampicin, Isoniazid and Pyrazinamide in a Single Step

**Published:** 2016

**Authors:** Karim Asadpour-Zeynali, Elhameh Saeb

**Affiliations:** *Department of Analytical Chemistry, Faculty of Chemistry, University of Tabriz, Tabriz, 51666 16471, Iran.*

**Keywords:** Simultaneous Determination, Chemometrics, Rifampicin, Isoniazid, Pyrazinamide

## Abstract

Three antituberculosis medications are investigated in this work consist of rifampicin, isoniazid and pyrazinamide. The ultra violet (UV) spectra of these compounds are overlapped, thus use of suitable chemometric methods are helpful for simultaneous spectrophotometric determination of them. A generalized version of net analyte signal standard addition method (GNASSAM) was used for determination of three antituberculosis medications as a model system. In generalized net analyte signal standard addition method only one standard solution was prepared for all analytes. This standard solution contains a mixture of all analytes of interest, and the addition of such solution to sample, causes increases in net analyte signal of each analyte which are proportional to the concentrations of analytes in added standards solution. For determination of concentration of each analyte in some synthetic mixtures, the UV spectra of pure analytes and each sample were recorded in the range of 210 nm-550 nm. The standard addition procedure was performed for each sample and the UV spectrum was recorded after each addition and finally the results were analyzed by net analyte signal method. Obtained concentrations show acceptable performance of GNASSAM in these cases.

## Introduction

Broadly speaking, univariate calibration methods are much better understood than multivariate calibration methods. Univariate data can be presented as plot of analyte concentration versus analyte signal and slope of the calibration graph is a measure of the sensitivity of the analytical method (1). Univariate methods need high selectivity for signal of an individual chemical species, but this selectivity is spoilt when there are signals other than that of analyte so that the interfering signals could not be vanished with background correction methods. Thus need arises for methods that use several variables instead of a single variable. Multivariate methods of calibration use several variables rather than a single variable and are among important methods of chemometrics that are use for determining a chemical species in the presence of other ones (2-5). During the past years the new concept of net analytesignal (NAS) has been introduced by Lorber (6). The concept focuses on calculating a signal, called net analyte signal, from the instrumental signal obtained from a group of several chemical species, which is purely related to gust one of the species under study. NAS of an analyte is that part of the spectrum of the analyte, which is orthogonal to the vector space spanned by spectra of the other components of the sample. Standard addition method based on NAS has been developed and found many applications (7-12). Four NAS-based methods have been developed and reported in the literature for multicomponent analysis: (I) hybrid linear analysis (HLA), which can be applied to a very accurately measured pure spectrum of analyte; (II) the HLA method developed by Xu and Schechter, which uses all the factors for prediction in order to build a method free from optimum factor estimation; (III) the HLA method developed by Goicoechea and Olivieri, which extracts interferents’ subspace with removing the analyte portion; and (IV) other NAS-based multivariate calibration methods, in which the vectors of the NAS of mixtures are used as input for other multivariate calibration methods, such as classical least squares, principal component regression (PCR), and partial least squares regression (PLS)(7-10). Recently, our research group have reported a standard addition method based on NAS as a simple method for applying NAS concept for simultaneous determination of chemical species with overlapping spectra or voltammograms, which direct simultaneous determination of them is impossible (3-5, 13). Very recently this method was used for analysis of chromatographic data (14). The method is capable of determining concentration of the analyte in the presence of interfering species of known identity. The method is also applicable with various instrumental analysis methods (4, 5, 13, 15). Having advantages of both NAS and standard addition methods, the method can determine concentration of the analyte in a single standard addition step. Multivariate calibration methods include two steps: calibration step and prediction step, while NASSAM only includes a single standard addition step. Using NASSAM, both of the two steps of multivariate methods are not needed (5). In 1979 Saxberg and Kowalski made a development in the standard addition method for multivariate data and called it generalized standard addition method (GSAM). 

**Figure 1:**
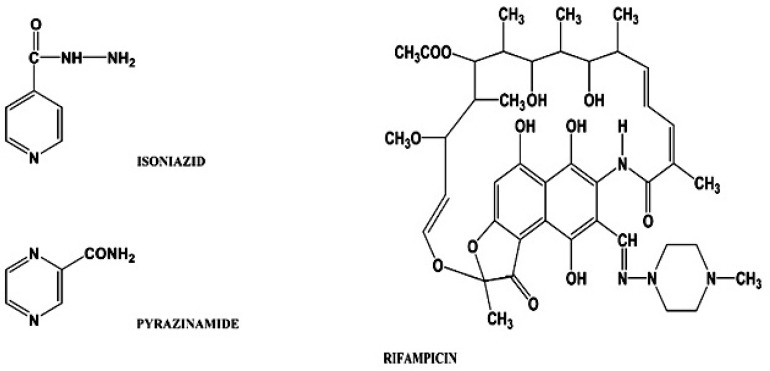
Structure of isoniazid, pyrazinamide and rifampicin

**Figure 2:**
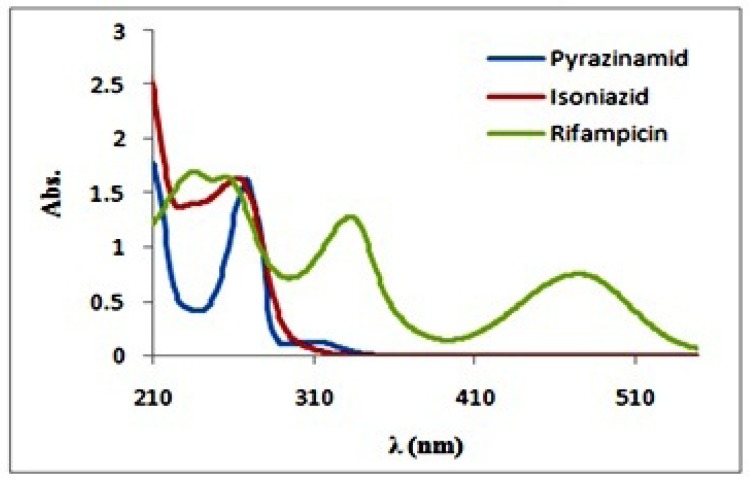
Absorption spectra rifampicin, isoniazid and pyrazinamide (C=30 mg/L

**Figure 3:**
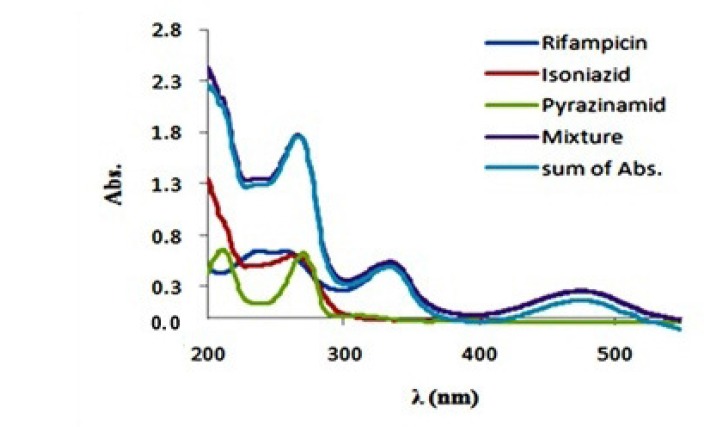
Absorbance (Abs.) spectra of rifampicin (15 mg/L), isoniazid (15 mg/L), pyrazinamide (15 mg/L) and their mixture in theoretical (Sum) and optimum experimental (Mixture) conditions

**Figure 4.**
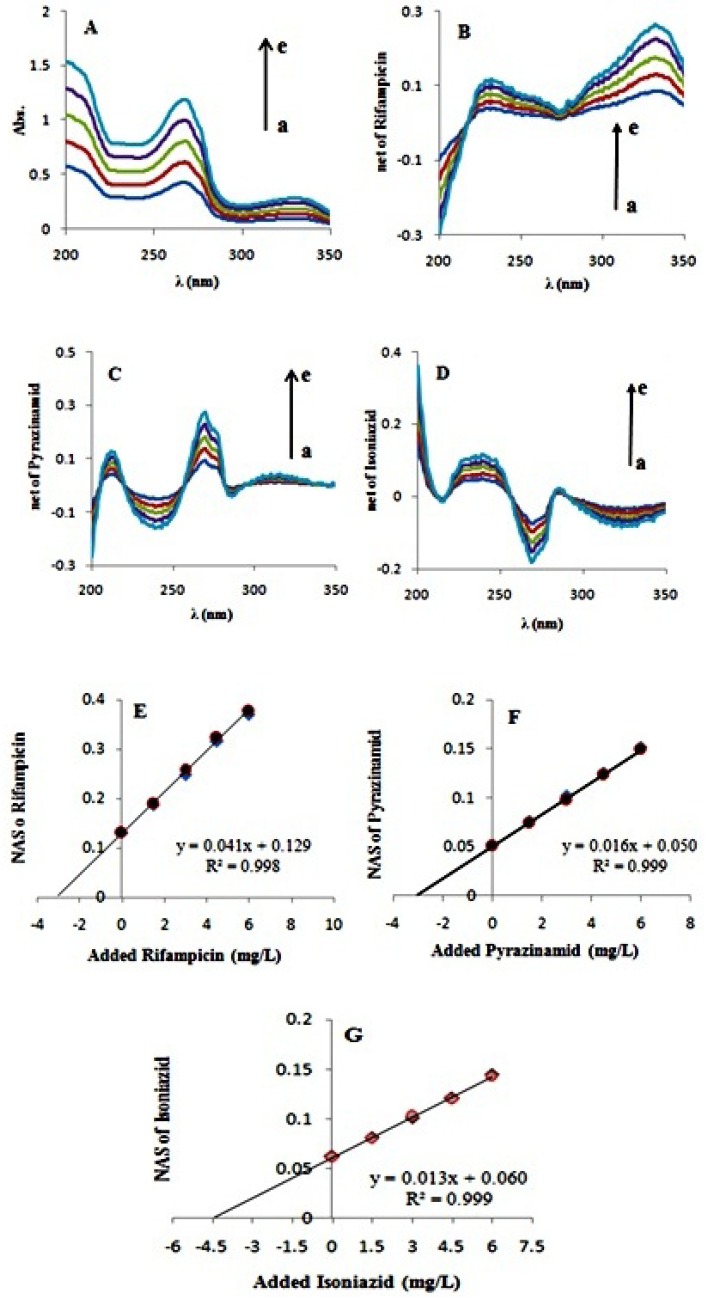
A) Absorption spectra of ternary synthetic mixture of antituberculosis drugs (a) before and (b-e) after addition of 1.5,3,4.5 and 6 mg L-1 of rifampicin, isoniazid and pyrazinamide; B) Net analyte signal of rifampicin (a) before and (b-e) after standard addition;

**Table 1:** Concentration of each analyte in synthetic sample

**Concentrations (mg/L)**		
**Rifampicin**	**Isoniazid**	**Pyrazinamide**
3	4.5	3

**Table 2:** Concentrations (mg/L) of added standards in each step of standard addition procedure

	Sample		
Observations	Rifampicin	Isoniazid	Pyrazinamide
**a**	0	0	0
**b**	1.5	1.5	1.5
**c**	3	3	3
**d**	4.5	4.5	4.5
**e**	6	6	6

**Table 3:** Results of analysis of isoniazid, pyrazinamide and rifampicin ternary mixture

**Analyte**	**Concentration (mg/L)** ** (standard deviation)**	**Error %**	**λ (nm)**
Rifampicin	3.06 (0.05)	2.0	250-300
Isoniazid	3.08 (0.02)	2.7	281-350
Pyrazinamide	4.46 (0.02)	0.9	270-350

**Table 4:** Analytical data from the calibration graphs for the determination of isoniazid, pyrazinamide and rifampicin by spectrophotometry

**Parameters**	**Rifampicin**	**Isoniazid**	**Pyrazinamide**
Equation of calibration curve	y = 0.396x - 0.043	y = 0.367x – 0.002	y = 0.135x + 0.028
R^2^	0.999	0.998	0.999
SD,** mg/L**	0.058	0.057	0.029
LOD, **mg/L**	0.581	0.523	0.682
Selectivity	0.0630	0.0882	0.0660
Sensitivity,** L/mg**	0.0424	0.0130	0.0168

**Table 5:** Results for analyses of rifampicin, isoniazid and pyrazinamide in pharmaceutical formulations by GNASSAM

**Analyte**	**No.**	**Added, mg/mL**	**Found, mg/mL**	**Mean**	**SD**	**Recovery, %** **(for mean)**
Rifampicin	1 2 3	2.00 2.00 2.00	1.91 1.90 1.81	1.87	0.05	93.50
Isoniazid	1 2 3	2.50 2.50 2.50	2.52 2.54 2.51	2.52	0.01	100.80
Pyrazinamide	1 2 3	3.00 3.00 3.00	3.02 2.97 2.98	2.99	0.02	99.67

The normal standard addition method assumes that for any one analyte in a sample there is an analytical sensor which responds to that analyte and no other component (i.e. interferences) in the sample. The generalized standard addition method (GSAM) provides a means of detecting interference effects, quantifying the magnitude of the interferences, and simultaneously determining analyte concentrations. In this method standards of analyte and interferent should be added consecutively. Generalized standard addition method was developed for analyzing multi-component mixtures and the method does not need a signal being completely analyte-selective. The method requirements just include: the signal can be zeroed (i.e. zero response when the analyte concentration is zero), and number of analytical sensors is no less than number of analytes. In the present article we present generalized net analyte signal-standard addition method (GNASSAM), which is in fact a version of NASSAM that with a single addition of a mixture of standard solutions, all of the analytes are determined (16).

We aim at using GNASSAM for determining ternary mixtures of antituberculosis drugs found in pharmaceutical formulations. One of the major difficulties in determination of coexisting drugs or drugs that are consumed simultaneously is our inability to measure them directly due to the overlap observed in their relevant signals. Regarding that all of the three drugs, rifampicin, isoniazid, and pyrazinamide, are used together for treating some particular disease, their simultaneous determination is of importance. Tuberculosis is a worldwide pandemic infectious disease, which annually causes millions of people to die (17). The treatment of tuberculosis is a worldwide problem. The structures of rifampicin, isoniazid and pyrazinamide are reported in Figure 1 (18). Rifampicin, isoniazid, and pyrazinamide are all among active bacterial antituberculosis drugs (19), which are prescribed for initial 2-months of the treatment course of pulmonary tuberculosis (20-21). Rifampicin and isoniazid are important antibiotics, which are widely used in treatment of tuberculosis and other infectious disease (22, 23). Rifampicin and isoniazid have been categorized as first-line drugs in dealing with tuberculosis. These drugs are highly effective, can be administered orally and tolerated well, and are of minimal toxicity. They are prescribed worldwide and rest of the antituberculosis drugs, such as pyrazinamide, is second-line antituberculosis drugs (24). Several fixed combination preparations of rifampicin, isoniazid and pyrazinamide have been offered for drug market. Recently many methods have been reported for determination of ternary mixtures of these compounds; e.g. reverse phase-liquid chromatography (18), Liquid chromatographic determination of isoniazid, pyrazinamide and rifampicin from pharmaceutical preparations and blood (19), multivariate spectrophotometric calibration (20), differential pulse polarography in conjunction with partial least squares (23), simultaneous spectrophotometric determination of rifampicin, isoniazid, and pyrazinamide by partial least squares combined with a modification of hybrid linear analysis calibration (24), and high-performance liquid chromatography after simultaneous extraction of the compounds from plasma (25). Most of the methods being used for determination of ternary mixtures of analytes need pretreatment and a preliminary extraction step. In the present work we used a method based on NAS with no need for any preliminary separation step, which is rapid and does not need large samples (26). 

The proposed method was applied for determination rifampicin, isoniazid and pyrazinamide in ternary mixtures as a model system.

## Experimental

*Theory*

Conventional notation has been used throughout the following discussion. Boldface capital letter is used for matrix, boldface lower case for column vector, and lightface lower case italic for scalar. The superscript T designates the operation of vector or matrix transposition, and superscript + denotes the pseudoinverse of a nonsquare matrix. The digitized spectrum is referred to as a spectrum vector, or simply as a vector, while a spectrum vector of a pure component is called a component vector. The following matrixes and vectors will be used: an m × n data matrix R composed of the calibration responses of m samples at n wavelength; an n × 1 vector s_k_, containing the pure spectrum of analyte k at unit concentration; and an m × 1 vector y_k_ of calibration concentrations of analyte k. Here, we briefly refer to NAS-based methods. The NAS for analyte *k(r*^+^_k_*)* is defined as the part of its spectrum that is orthogonal to the space spanned by the spectra of all other analytes and is given by the following equation:

(1) $$r_{k}^{+}=\left[I-R_{-k}{\left(R_{-k}\right)}^{+}\right]R=P_{\mathrm{NASK}}R$$

where R is the spectrum of a given sample, *I* is an n × n unitary matrix, and R_–k_ is an n × A column space spanned by the spectra of all other analytes except k. When R is the profile s_k_ of pure k at unit concentration, Equation 1. becomes *s*_k_^+^ = *P*_NAS,k_s_k_, (R_-k_)^+^ is the pseudoinverse of R_-k_, A is the number of factors used to build the model, and P_NAS_,_k_ is an n × n projection matrix that projects a given vector onto the NAS space; *r*^+^_k_ can be used for quantification of the analyte.

In binary and/or ternary mixtures when the interferences are known, the R_-k_ is the space of interferents and *r*^+^_k_ can be calculated for the analytes easily. The norm of the *r*^+^_k_ can be used to construct a univariate calibration model, where this parameter is plotted against the analyte concentration and a linear relationship is observed.

Generelized net analyte signal standard addition method (GNASSAM): The background and theory of the GNASSAM have been discussed in full detail in previous paper (27). Hence, in generalized net analyte signal standard addition method only one standard solution was prepared for all analytes. This standard solution contains a mixture of all analytes of interest, and the addition of such solution to sample, causes increases in net analyte signal of each analyte which are proportional to the concentrations of analytes in added standards solution.

The GNASSAM is the only general method that can simultaneously correct for interference and matrix effects. The method can be applied using as little as one spike (addition) per analyte/interference pair and need not represent an inordinate amount of additional labor (28).

*Apparatus and Software*

All spectrophotometric measurements were carried out on Analytik-Jena (SPECORD 250-222P173) spectrophotometer, using 1.0 cm quartz cells. All spectra were saved in ASCII format, and then all data were converted to an Excel file, in the next step they were converted to MATLAB formats. All spectrophotometry data were transferred to a personal computer (PC) for subsequent manipulation. The pH measurements were performed by means of a digital pH-meter (Metrohm, Riverview, FL; Model 744). Data processing was executed by MATLAB^®^ software. Computer programs required for NAS calculation was written in MATLAB^®^.

**Reagents and Solutions**

All experiments were performed with analytical grade chemicals and solvents. The water utilized in all studies was deionized. 100 mg/L Stock solutions of rifampicin, isoniazid and pyrazinamide were prepared by dissolving the appropriate amounts of the chemicals in deionized water and the prepared solutions were stored in refrigerator at 4 °C. The stock solutions were appropriately diluted daily for doing experiments.

The pharmaceutical formulations used for assessing applicability the method included: 300 mg rifampicin tablets, 300 mg isoniazid tablets, and 500 mg pyrazinamide tablets, which were manufactured by Abidi, and Hakim pharmaceutical companies (Tehran, Iran) and were purchased from local drug stores.

Britton-Robinson buffer solutions, with pH = 1-12, were prepared by mixing an acid mixture (composed of H_3_PO_4_, H_3_BO_3_, and HC_2_H_3_O_2_) with 0.2 M NaOH, all in appropriate proportions. HCl and NaOH solutions were used respectively for buffering pH at 1-2 and 12-13. Buffer solutions were prepared with the aim of investigating the effect of pH on the analytical system.

*Procedure*

A synthetic ternary mixture of Rifampicin, Isoniazid and Pyrazinamide in deionized water was prepared as sample. Composition of this sample is given in Table 1.

At first the spectrophotometric absorption of sample was recorded between wavelengths 200 to 550 nm with 0.5 nm intervals. Standard addition was performed on sample and the absorption spectrum of sample was recorded after each addition step. Table 2. shows the amount of added concentration of each analyte into the sample. All observations were recorded and after calculation of NAS of each observation, diagram of NAS versus the concentration of added standards were plotted. The Concentration of each analyte in the sample was determined from length of the abscissa-intercept or x-intercept of the linear graph. Standard addition method, replicates at least 3 times and the average and standard deviation of results were reported.

## Results and Discussion

*Absorption spectra*

The absorption spectra of isoniazid, pirazinamide andrifampicin are plotted in Figure 2. As we can seein the ﬁgure, the absorption spectra show absorptionmaxima located at 335 and 475 nm for rifampicin‚, 279 nm for isoniazid and 269 nm for pyrazinamide. Hence, spectral overlap between their absorption spectra is evident and therefore they cannot be simultaneously determined by univariate methods without prior chemical separation. Absorption spectra of rifampicin, isoniazid and pyrazinamide did not change over the pH range of 5–7.5 (The plots were not reported here). At first the preliminary necessity for applying any bilinear method was examined and the bilinear behavior for spectrophotometric data of the antituberculosis drugs was confirmed. For this purpose, the linearity and additivity of spectrophotometric responses were investigated. The individual spectra, mixtures, and sum of the spectra for rifampicin, isoniazid, and pyrazinamide were plotted. Figure 3. shows the individual spectra, mixtures, and sum of the spectra for rifampicin, isoniazid and pyrazinamide. According to Figure 3. there are no interactions between analytes, and the signals have very good additive properties. Absorption spectra of these substances were not changed by varying pH in the range 5-7.5 and therefore this pH range was chosen as the optimum range.

*Determination of Rifampicin, Isoniazid and Pyrazinamide by GNASSAM*

In the present work we introduced a method with no need for any preliminary separation step, which is rapid and does not need large samples. The GNASSAM is also based on the concept of net analyte signal, which was developed by Lorber. The generalized standard addition method (GSAM) is a method of multicomponent analysis which provides a means of detecting the Interference effects, quantifying the magnitude of the interferences, allowing the use of the most sensitive wavelengths for all analytes, and simultaneously determinlnganalyte concentrations. We aim at using GNASSAM for determining ternary mixtures of antituberculosis drugs found in pharmaceutical formulations.

One ternary synthetic mixture of antituberculosis drugs was investigated. In this ternary mixture every three contributions are considered as analytes. For determination of analytes concentrations, known amounts of a mixture standard solution (of every three analytes) successively added to the sample and the UV spectra of mixture were recorded. A wavelength selection step was used to select optimum range of wavelengths. For this purpose the correlation coefficient of the fitted straight line in standard addition diagram and also the value of EI function were considered. By adding the three antitubercuosis drugs into a mixture of them, absorption of all of the three species increase simultaneously (Figure 4-A). By adding standard solutions of the antituberculosis drugs, rifampicin, isoniazid, and pyrazinamide, their absorption vector enlarges simultaneously as well as their net analyte signal (NAS) (Figures 4-B, 4-C, and 4-D). According to what was mentioned above, net analyte signal is the portion of data which is orthogonal to the vector space of interfering species. Here we considered three cases: (i) isoniazid and pyrazinamide were assumed as interferens and rifampicin was determined in the presence of them; (ii) rifampicin and isoniazid were regarded as interfernts and pyrazinamide was regarded as the analyte; and (iii) isoniazid was to be determined in the presence of the other two species. Norm of the net analyte signal is increased upon increasing concentration of the added standard. By plotting norm of the NAS versus concentration of the added analyte standards, the analyte concentration (i.e. rifampicin) in the presence of the interferents (i.e. isoniazid and pyrazinamide) can be obtained (Figure 4-E). Figure 4-F. depicts norm of NAS of the analyte (i.e. pyrazinamide) versus concentration of the added analyte standards, where concentration of pyrazinamide is determined in a mixture of the analyte an interferents (i.e. rifampicin and isoniazid). Figure 4-G. shows a similar graph with isoniazid as the analyte and rifampicin and pyrazinamide as the interferents. As can be seen in all of theFigures 4-E, 4-F, and 4-G, norm of each species is increasing due to the simultaneous addition of the three standards. By GNASSAM concentrations of analytes were simultaneously obtained with a single step procedure. In other word, calibration and prediction steps that are usual in multivariate calibration methods were eliminated in GNASSAM. As can be seen from 4-E, 4-F, and 4-G the position of the standard addition plot is only dependent on the analyte concentrations and is independent of other interferent concentration. Selected wavelength ranges and the calculated concentrations were summarized in Table 3.

*Figures of merit*

Selectivity is regarded as the amount of final overlap, which determines the part of a spectrum which cannot be observed due to overlap (5, 6). Selectivity assigned to orthogonal spectra with no overlap equals 1 and the value assigned to those that overlap fully is equal to 0.

Selectivity is obtained from equation 2 by dividing by spectrum of the sample to be analysed.

$\mathrm{SEL}=\frac{\left\|S_{k}^{*}\right\|}{\left\|S_{k}\right\|}$

(2)

designates spectrum of the pure analyte when it is present at unit concentration.

Sensitivity (5, 6) is one of the figures of merit that shows the rate of instrumental response with the change of concentration. Sensitivity in univariate calibration methods is regarded as slope of the calibration curve and this requires a linear relationship between regression coefficient and sensitivity. Note that in reverse calibration methods one can even calculate sensitivity using the linear relationship. With the method presented here for calculation of NAS, one can calculate sensitivity vector for each calibration sample.

Sensitivity is calculated by equation 3(8-10).

(3) $$\mathrm{SEN}=\frac{\left\|r_{k}^{*}\right\|}{\left\|C_{k}\right\|}=\left\|S_{k}^{*}\right\|=\frac{1}{b_{k}}$$

$r_{k}^{*}$ is the net signal of the mixture, $c_{k}$ is analyte concentration, $S_{k}^{*}$ is net analyte signal, and b_k_ is regression coefficient.

Limit of detection (LOD) (5, 6) is an important parameter in analytical methods such as atomic absorption spectrometry and mass spectrometry, where calibration curves may be extended to the background level of the instrument.

LOD can be calculated by equation 4: 

(4) $$\mathrm{LOD}=\frac{3\left\|\varepsilon \right\|}{\left\|S_{k}^{*}\right\|}=3\left\|\varepsilon \right\|\left\|b_{k}\right\|$$

Where$\left\|\varepsilon \right\|$ designates error of determination. may be calculated by recording absorption spectra of several blank samples followed by calculating NAS of each sample and finally calculating the relevant standard deviation. The standard deviation is taken as an approximation of$\left\|\varepsilon \right\|$.

Selection of sensors: To select optimum range of sensors (wavelengths), an error indicator (EI) as a function of a moving window was calculated for each sample using information of the net analyte signal regression plot (NASRP) (25). The NASRP is a plot of the elements of the sample vector $S_{k}^{*}$versus those of $S_{k}^{*}$ and should fit a straight line through the origin, with random residuals and slope c_k_. Large and correlated residuals in this plot reveal discrepancies between the measured profile and the model and, possibly, bias in the estimated concentration. The expression for EI used in the present context is:

(5) $$\mathrm{EI}=\frac{S_{d}}{\left\|r^{*}\right\|}={\left(1+\frac{N^{2}S_{d}^{2}}{4{\left\|r^{*}\right\|}^{2}}\right)}^{0.5}$$

where is the standard deviation from the best fitted straight line to the NASRP and *N* is the number of points in the latter plot. The selected wavelengths were reported in Table 3.

In Table 4. the calibration graphs, calculated selectivity & sensitivity in ternary mixtures and LOD for analytes have been summarized.

*Application to Synthetic Samples*

In order to determine the drugs in the tablets simultaneous standard addition method was performed. A fixed volume of solution of rifampicin, isoniazid, and pyrazinamide tablets were added into five 10 mL volumetric flasks. Then standard solutions were added simultaneously and the absorption spectrum of each of the obtained solutions was recorded. Data were saved in .txt format and we then transformed into Excel and MATLAB format and norm of NAS of rifampicin, isoniazid, and pyrazinamide were separately plotted versus concentration of the added standard. Concentration of each analyte in tablet was determined from x-intercept of its relevant graph. The result of analysis of ternary mixture of rifampicin, isoniazid, and pyraziamide tablets were obtained. Assessing accuracy of the method was achieved by spiking the real samples and investigating percent recoveries because there is no standard method for performing such a chemical analysis. Standard addition to the solutions was performed. A graph of NAS for each of the three drugs was plotted. Table 5. shows the obtained results for three replicate determinations of the analytes. Considering the standard deviations and percent recoveries of the spiked amounts, one can conclude that the method offers satisfactory accuracy.

## Conclusion

Determination of rifampicin, isoniazid and pyrazinamide has been accomplished from spectrophotometric data with a novel method based on the NAS and generalized standard addition method. The analysis can be carried out without preliminary separation steps. The proposed method eliminates the calibration and prediction steps of multivariate calibration methods, and in the presented method, simultaneous addition of standards of different chemical species, and therefore single-step simultaneous determination of species, is possible. The proposed method (GNASSAM) shows some advantages in comparison with other methods e.g. performing only one standard addition procedure for analysis of multi-analyte samples and so it causes to save time and sample size and ability to analysis of data even when the concentrations are linearly dependent. The proposed method is simple, rapid, and in-expensive and needs no pre-treatment step; so it can be recommended for routine analysis of mixtures of analytes with spectral overlap.
